# A Portable ‘Plug-and-Play’ Fibre Optic Sensor for In-Situ Measurements of pH Values for Microfluidic Applications

**DOI:** 10.3390/mi13081224

**Published:** 2022-07-30

**Authors:** Rahul Kumar, Hien Nguyen, Bruno Rente, Christabel Tan, Tong Sun, Kenneth T. V. Grattan

**Affiliations:** 1 Department of Electrical and Electronic Engineering, University of London, London EC1V 0HB, UK; rahul.kumar@city.ac.uk (R.K.); hien.nguyen@city.ac.uk (H.N.); bruno.rente@city.ac.uk (B.R.); t.sun@city.ac.uk (T.S.); k.t.v.grattan@city.ac.uk (K.T.V.G.); 2 School of Physics, Engineering and Computer Science, University of Hertfordshire, Hatfield AL10 9AB, UK

**Keywords:** microfluidics, pH sensor, optical fibre sensor, fluorescent sensor

## Abstract

Microfluidics is used in many applications ranging from chemistry, medicine, biology and biomedical research, and the ability to measure pH values in-situ is an important parameter for creating and monitoring environments within a microfluidic chip for many such applications. We present a portable, optical fibre-based sensor for monitoring the pH based on the fluorescent intensity change of an acrylamidofluorescein dye, immobilized on the tip of a multimode optical fibre, and its performance is evaluated in-situ in a microfluidic channel. The sensor showed a sigmoid response over the pH range of 6.0–8.5, with a maximum sensitivity of 0.2/pH in the mid-range at pH 7.5. Following its evaluation, the sensor developed was used in a single microfluidic PDMS channel and its response was monitored for various flow rates within the channel. The results thus obtained showed that the sensor is sufficiently robust and well-suited to be used for measuring the pH value of the flowing liquid in the microchannel, allowing it to be used for a number of practical applications in ‘lab-on-a-chip’ applications where microfluidics are used. A key feature of the sensor is its simplicity and the ease of integrating the sensor with the microfluidic channel being probed.

## 1. Introduction

The accurate and rapid measurement of the pH value of a solution is important in determining its chemical condition, and hence such measurements are widely needed by and used in industry [1]. Measuring the pH is essential not only for finding the key characteristics of a substance but also in the management of many chemical reactions. Further, pH measurement is used in nearly all industries that deal with water contamination and purity, not only the chemical industry but also public organizations, including the agriculture and manufacturing industries.

Examples are seen in various related sectors [1], including health care [2,3,4,5,6], the food processing industry [7,8,9,10], textile mills [11,12], wastewater management [13,14,15] and environment monitoring [16,17,18]. In the field of microfluidics, pH measurement is a critical parameter for cellular culture environments along with many other biomedical applications from bioreactors to on-chip analysis platforms to full lab-on-a-chip applications [19].

To cater for this widespread requirement of easy, usually low-cost pH measurement across a range of diverse environmental conditions, several different types of pH sensors based on techniques, such as potentiometric and capacitive changes [20,21,22], micro-electrical mechanical systems (MEMS) [23], surface acoustic waves [24,25], localized surface plasmon resonance [26,27,28,29,30], fluorescent dyes [31,32], carbon nanotubes [33,34] and Complementary MOS (CMOS) [35] has been reported in recent years. 

Some of these techniques can be employed in an optical fibre-based configuration, as that offers advantages (in contrast to the electrical counterparts) in terms of immunity to electromagnetic interference and resistance to harsh and corrosive chemical environments, also allowing a remote sensing capability [36,37,38]. In several of the techniques mentioned above, optical fibres have been used as a passive element (simply to transport light to and from the sensing head [39]), whereas in many others they play an active role in the sensing process itself [40,41,42]. One of the main issues faced when developing pH sensors for microfluidics is the small volume of the liquid used, coupled with a constant flow within the device, which makes any direct pH measurement challenging.

This field of research and development in pH monitoring is an active one and has been for many years. When introducing a new sensor device, it is important to assess the strengths and weaknesses of those that have been reported before and to build on that—the recent paper by some of the authors have done that [1] (and this is not reproduced here). In summary, a brief overview comparison of the key features of electronic and optical fibre sensors, applied for measuring pH, can be used to highlight both their main characteristics and advantages/disadvantages, which then is helpful in the design of the sensor discussed in this work. 

For example, a range of commercial pH sensors has been designed to work in the world’s most extreme liquid analysis applications [43], for tough industrial applications. This sensor has been optimized to create measurement solutions for extreme applications, such as precious metal refining (gold, copper, nickel and zinc), or for titanium dioxide production, ammonium nitrate, solvent extraction and industrial wastewater applications. By comparison to the optical fibre sensor discussed here, the commercial device [43] requires a solid-state reference, combined with a glass electrode—new types of toughened glasses have been used when conventional glass or lab-grade electrodes are not suitable. 

The optical fibre approach avoids the need for such electrodes in the measurement process. Thus, in spite of considerable work to date worldwide, and indeed over several decades, there is still considerable scope for improvement of the device in terms of their suitability for long-term use in real-life applications. Many of the previously reported optical sensors demonstrated in laboratories either are not readily portable for use in the field [26,27,37,44], require to be used in darkness [26,27,39,44] (to avoid interference from the ambient light) or in a static liquid [27,37,39]: all these render them less than optimal for use in most industrial situations.

In this investigation, which aims to validate the approach put forward and which is strongly application-focused, a study of these problems that restrict in-the-field use and are associated with some of previously developed and reported optical pH sensors are addressed. Acrylamidofluorescein (AAF) dye-based optical fibre sensors have been designed for a variety of real-world applications, including the important measuring of pH of a flowing liquid in a microfluidic channel. 

Liao et al. [45] provided an interesting review paper, and this provided some useful background through an overview of the state-of-the-art. Further, Moradi et al. [46] referenced the use of a polymethylmethacrylate (PMMA) mixing device actively to produce solutions at different pH values using HPTS. Design-wise, this has some similarities to the work reported herein, in the sense that it requires a larger detection chamber and they used two detectors and fluorescence-based detection of pH. 

Zamboni et al. [47] used a different but interesting technique of pH measurement. The sensor is inherently built into the silicon device itself but created a pH sensor, which differs from the approach herein, in that it is not optical. This does show that different methods can be used, and this gives the user maximum flexibility in the choice of method. 

In addition, Pinto et al. [48] employed a colourimetric technique using an indicator dye—this is also an interesting but different technique using photodetectors. In their paper, Budinski et al. [49], in their paper, detailed the manufacturing of a glass sensor that uses optical absorbance at the reagent-specific wavelength to determine pH, while Elmas et al. [50] discussed a photometric sensing method in which the chip is made in glass for a clear pathway. Thus, there is considerable work in the literature on which to build as well as to point in new directions that can be exploited herein.

This design discussed in this work was created to overcome some of the issues seen with prior research and thus to take advantage of the excellent work done by others (discussed above). In the design proposed herein, we proposed that not only would the sensor readings be conveniently collected under ambient light conditions but also a perylene red dye would be added to the probe as an ‘internal reference’ to allow a correction to be made to minimize the influence of important, potentially interfering external factors to the instrument reading, such as light source intensity fluctuations and temperature changes. 

The microfluidic channels (from ‘lab-on-a-chip’ applications) considered here were used in this demonstration because of their increased usage in several important, practical areas where pH measurement is needed, such as chemical analysis [51,52] and biological analysis [53,54,55,56]. Since such microfluidic channels can readily be realized at low cost and show important intrinsic advantages (in particular, needing only a small volume sample of reagent), they are key to creating effective, real-time point-of-care devices, including the important ‘lab-on-a chip’ devices that are being widely used today [51,52,53,54,55,56,57,58,59]. The major novelty and key insight in this work can be summarized as providing a demonstration in the probe developed of a portable, plug-and-play microfluidic pH sensor, working well in ambient light conditions and providing an additional device available to the user.

## 2. Principle of Operation

**Figure 1:**
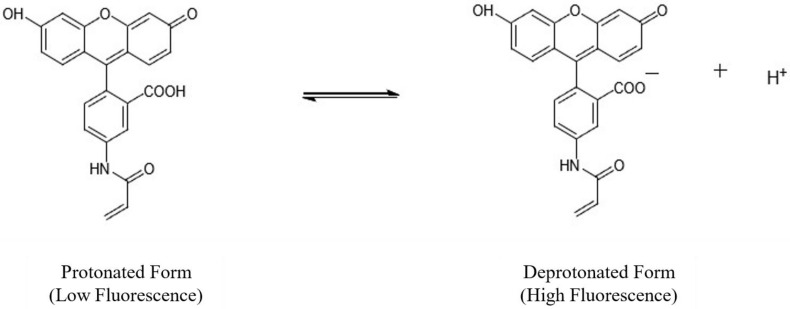
Schematic showing the equilibrium of protonated and deprotonated forms of the AAF dye in solution.

The sensor developed uses the protonation–deprotonation of fluorescent AAF dye immobilized at the tip of optical fibre, represented as HA, in an aqueous solution—this being the reason that the pH-induced intensity change is observed. The fluorescence intensity of the deprotonated (basic) form is greater than that of the protonated (acidic) form, and this reaction in its equilibrium form, depicted in Figure 1, can be represented by:

(1) $$HA⇌H^{+}+A^{-}$$

The relationship between the concentration of the protonated and the deprotonated forms and the value of pH is governed by the Henderson–Hasselbalch reaction, given in Equation (2):

(2) $$pH=pK_{a}+\log\frac{\left[A^{-}\right]}{\left[HA\right]}$$

where [*A^−^*] and [*HA*] are the concentrations of the deprotonated and protonated forms of the fluorescence dye, respectively, and *pK_a_* is an acid–base constant. Since the concentrations of the deprotonated and the deprotonated forms are directly proportional to the intensity of the fluorescence observed, Equation (2) can be written in terms of the observed intensities as shown in Equation (3):

(3) $$pH=pK_{a}+\log\frac{F-F_{min}}{F_{max}-F}$$

where *F_min_*, *F_max_* and *F* are the fluorescence intensity of fully protonated system, the fluorescence intensity of fully deprotonated system and the measured fluorescence of the system. In the case where a reference signal is used, the fluorescence intensity ratio (R) can be determined by dividing the signal intensity by the reference intensity (*F_ref_*) (as given in Equation (4)). In the present work, the fluorescence intensity from a perylene red dye was used as a reference because it has a convenient, overlapping excitation band with the AAF dye, thus a single light source is sufficient for the excitation of both chemicals. The presence of *F_ref_* then can be used to modify Equation (3) to Equation (5) as shown below:

(4) $$Ratio\;\left(R\right)=\frac{F}{F_{ref}}$$

(5) $$pH=pK_{a}+\log\frac{R-R_{min}}{R_{max}-R}\;$$

where $R_{min}$ and $R_{max}$ are, respectively, the minimum and maximum ratio obtained. Thus, R can be written as:

(6) $$R=\frac{R_{min}+R_{max}\times 10^{\left(pH-pK_{a}\right)}}{10^{\left(pH-pK_{a}\right)}+1}$$

It can be seen that Equation (6) shows the ‘S-shaped’ relationship between the fluorescence ratio, *R*, and the value of the pH, which is centred around the *pK_a_* value. This equation is used to calibrate the response of the sensor in a static liquid, as well as in the microfluidic channel.

As it will be shown later, the dye (AAF + perylene red) becomes coated on the side of fibre during the functionalization process; however, it should be noted that only the dye present on the tip of the optical fibre participates in the creation of a pH-sensitive signal—not the dye present on the side of the fibre—as the sensing mechanism is not, in this case, based on the modification of the evanescent wave ‘tail’ (a technique previously reported in the operation of several evanescent wave-based sensors [60,61,62]). Thus, the optical fibre is only acting as a passive element, i.e., carrying the light from the source to the dye and then from the dye to the spectrometer.

## 3. Experimental Section

### 3.1. Chemicals and Reagents

All chemicals were of analytical grade, purchased from Sigma-Aldrich, UK (except perylene red, which was purchased from Kremer Pigmente, Germany). All solvents used were of HPLC or analytical grade from Fisher Scientific UK. All aqueous solutions were prepared using deionized water.

### 3.2. Synthesis of the Fluorescent Dye and Optical Fibre Probe Preparation

AAF was prepared from fluoresceinamine according to the procedures reported in the literature [63], and perylene red was added to the pre-polymerization mixture to provide an ‘internal reference’. After synthesis of the fluorescent dye, it was immobilized on the surface of the optical fibre selected according to the method successfully employed by some of the authors in the development of other such probes (previously reported in the literature e.g., [39]). In summary, a 150 mm long polymer-clad multimode silica fibre, with a 1000 µm core diameter (FT1000UMT; Thorlabs, UK), was used as the substrate for the coating. 

The 1000 µm diameter multi-mode optical fibre gives a much greater coating area than single-mode communications-type fibre—with a typical diameter of around 5 µm— and in addition, provides the sensor with good mechanical strength. The polymer cladding from the optical fibre was removed, and it was (manually) polished using 5, 3 and 1 μm grit polishing sheet (LFG series, Thorlabs, UK) in that same sequence, to minimize any unwanted losses during light coupling. 

After polishing, one facet was glued to an SMA connector (11050A; Thorlabs, UK) and to another end, the dye-based coating was functionalized by immersing it in 10% KOH in isopropanol for 30 min, with subsequent rinsing in copious amounts of distilled water and dried with compressed nitrogen. Following that, it was treated in a 30:70 (*v*/*v*) mixture of H_2_O_2_ (30%) and H_2_SO_4_ (95% laboratory Reagent Grade) (Piranha solution) for 30 min, rinsed in distilled water for 15 min and dried in an oven at 100 °C for 30 min. 

This procedure leaves the surface with exposed hydroxyl groups, which facilitate the bonding of a silane agent. The fibre surface was then modified by silanizing for 2 h in a 10% solution of 3-(trimethoxysilyl) propyl methacrylate in dry ethanol. The fibre was washed with ethanol repeatedly in an ultrasonic bath. Subsequently, it was dried in an oven at 70 °C for 2 h. This procedure functionalizes the fibre surface with polymerizable acrylate groups.

The pre-polymerization mixture was prepared by dissolving AAF (4.0 mg, 0.01 mmol), perylene red reference polymer (2.5 mg), ethylene glycol dimethacrylate crosslinker (150.9 µL, 0.8 mmol), acrylamide co-monomer (10.0 mg, 0.14 mmol) and 2,2′-azobisisobutyronitrile initiator (1.1 mg) in 222 μL dry MeCN. The solution was purged thoroughly with argon for 10 min. A small volume of the solution was placed into a capillary tube using a syringe, and the distal end of the fibre was inserted. They were sealed quickly with PTFE tape and polymerized in an oven at 70 °C for 16 h. 

**Figure 2:**
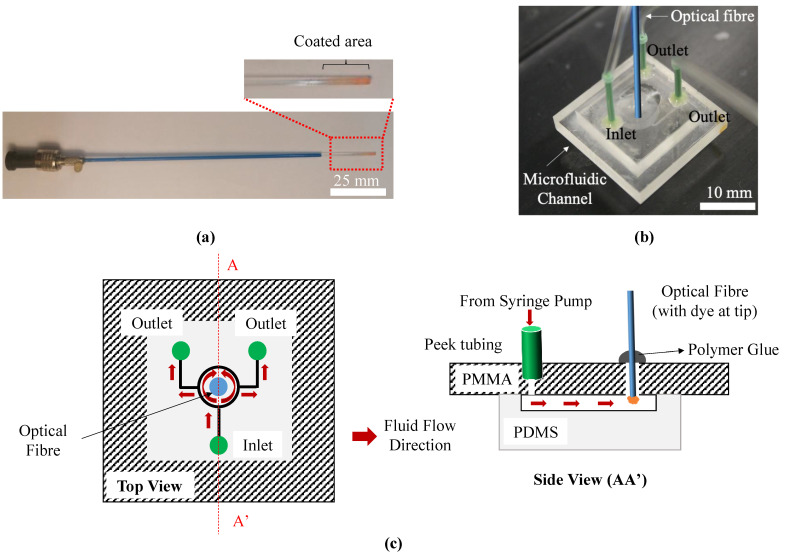
(**a**) Coated multimode fibre with a core diameter of ~1 mm. Inset shows a magnified version of the coated area. The coated area is fully inserted into the sensing chamber. (**b**) Fibres sealed in the microfluidic channel. (**c**) Top and side schematic view.

This procedure forms a polymer layer on both the cylindrical surface and the distal end surface of the fibre. The probe prepared by this procedure is shown in Figure 2a where it can be seen that the distal end of the probe shows a distinctive colouration due to the presence of the fluorophore. The sensor tip was washed repeatedly with MeOH-AcOH (8:2, *v*/*v*) in an ultrasonic bath, followed by the same procedure with MeOH alone to remove all unreacted materials and the excess amount of polymer formed, which was not directly bound to the fibre. The probe was then stored at room temperature in a dark box until needed for use in the experiments described below.

### 3.3. Fabrication of the Microfluidic Channel

The device consists of a circular sensing well, 1 cm in diameter and 100 µm in depth. The device consists of a single inlet port with two outlet ports to provide an alternative flow path in an event of a blockage due to trapped bubbles within the channel. The inlet and outlet channels are 200 µm wide and 100 µm in depth. The channel microstructure used in this work was manufactured ‘in-house’ using a rapid prototyping process previously reported by Johnston et al. [64] where, in summary, SU-8 2050 (A-Gas Electronic Materials, Warwickshire, UK) moulds were fabricated on silicon wafers. The silicon moulds provide a replication template for any future castings. 

PDMS structures were then cast from Sylgard 184 elastomer (Onecall Farnell, Leeds, UK) mixed in the standard 10:1 component ratio. All PDMS devices cast from the same SU-8 mould replicate the structures on the mould. The PDMS was then cured at 65 °C for 2 h and afterwards connected to a 5 mm poly (methyl methacrylate) (PMMA) sheet was used to close the fluid channels and provide fluid connectivity. The PMMA (Weatherall, Aylesbury, UK) was drilled to implement accurately located ‘through vias’ for inserting the locating tubing and the fibre optic sensor developed. 

All through-vias in the PMMA were drilled using a standard benchtop drill press as countersinks to ensure that any adhesives used to secure the tubing will not flow into the channel. The tubing used was 1.57 mm (1/16″) OD and 0.76 mm (0.03″) ID PEEK tubing (Cole-Parmer, Eaton Socon, UK). The vias were drilled in two parts, a 1 mm hole was first made through the PMMA bulk, followed by a 1.8 mm hole drilled halfway through the PMMA bulk.

The PDMS and PMMA components were then bonded by using a modification of the PMMA substrate using silane. The in-house protocol used was as follows. Clean, dry PMMA was exposed to UV-Ozone using a PSD-UVT system (Novoscan Technologies Inc., Ames, IA, USA) for 5 min. The PMMA was then silanized using aminopropyltriethoxysilane (APTES, 80 μL in a gas-tight 100 mL container) vapour for 1.5 h at 60 °C, at atmospheric pressure. The cooled PMMA substrate was immediately rinsed with isopropanol and dried with filtered nitrogen gas. Clean, dry PDMS was then exposed, bonding side up, to UV-Ozone for 3 min with the PSD-UVT system. The two treated components were carefully aligned and then brought together immediately. The composite device was then baked at 60 °C for 12 h to create a strong, irreversible covalent bond between the two materials.

### 3.4. Integration of Optical Fibre with Microfluidic Channel

The microfluidic device was designed and manufactured to permit the fitting of the optical fibre sensor into the sensing channel without the need for any additional sealing element. In the absence of any permanent fixture between the sensor and the microfluidic device, this allows the sensor to be extracted for reuse if needed.

The optical fibre used was carefully inserted into the 1.2 mm diameter hole drilled into the PMMA cover to ensure a snug fit. To avoid scratching the coating on the tip, a lab jack was used to position the fibre in place at a depth of 5 mm—the same as the thickness of the PMMA sheet and sealed with a polymer (Elastosil RT 601A, Wacker Chemie, Munish, Germany). Due to the size of the hole drilled, the fibre was able to sit tightly in the hole drilled, even without the use of the polymer. 

Whilst unnecessary, the decision to use RT601 A silicone rubber was taken to provide further security—to further secure the fibre so that it is not movable during experiments and to create a semi-permanent, water-tight seal around the fibre to prevent leakage. Using a flexible polymer, instead of permanent glue, enables a clean extraction of the sensor, if necessary. Elastosil sets to a shore hardness of 45, which is akin to the mechanical properties of rubber bands, hence, that it could be easily pulled apart, without damaging either the sensor or the microfluidic device, allowing both parts to be re-used, as necessary. 

Figure 2b,c, respectively, show the optical fibre sealed in the microfluidic channel and the top and front schematic views of the setup. One out of three through-vias was used as an inlet, connected to the syringe pump through the tubing while the other two were used as outlets. The optical fibre was inserted in the central chamber in such a way that its coated tip will be in contact with the flowing liquid. Such a setup can easily be modified for injecting two reagents (from two through-vias), and thus the measurement of the pH of the resultant solution in the central chamber.

### 3.5. Characterization Setup

**Figure 3:**
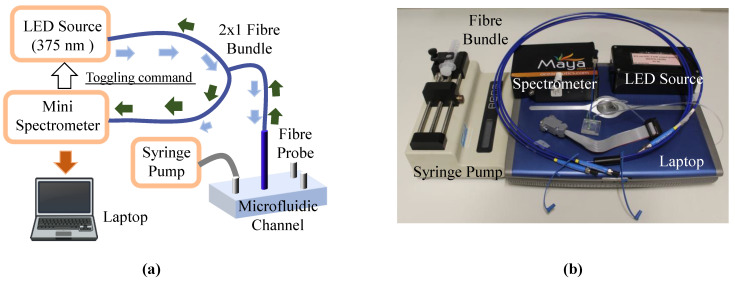
(**a**) Schematic of the experimental setup. Toggling of LED was controlled by sending pulse signal to electromechanical relay using a spectrometer. (**b**) Photograph showing several of the important components used in the setup.

The schematic of the setup used to investigate the performance of the sensor showing the key instruments needed for the characterizations carried out is shown in Figure 3a,b. As can be seen from the figure, the LED source (model number NS375L-ERLM; λ=395 nm; power = 3 mW) was coupled to one end of the 2 × 1 bundle (ϕ = 230 µm; Ocean Optics) with the help of a collimating and focusing lens (not visible in the figure as it was enclosed in the black LED source box), with a further end connected to a portable spectrometer (Maya-type 2000PRO; Ocean Insight, Wales, UK). 

The third (and the last) end, containing the other ends of the source and the ‘spectrometer fibres’ was connected to the optical fibre probe. It should be noted that the spectrometer is not strictly necessary (it was used here as one was available in the laboratory) but for a lower-cost option, a photodiode with two band-pass filters could have been used. This would further reduce the setup size and, of course, the cost. 

To evaluate its performance, the fibre probe was dipped into different solutions of known pH (this being pre-determined using a commercial pH sensor) to measure its response in static liquid: whereas for pH response in the microfluidic device, a single syringe infusion pump (model KDS 100; Cole-Parmer, Eaton Socon, UK) was used to regulate the flow rate of the solution (of known pH) into the channel. The sensor was tested for maximum and minimum flow rate, i.e., 6 mL/h and 509 mL/h, which can be achieved by the syringe infusion pump with 60 mL and 30 mL syringe, respectively. Figure 3b also shows the overall size of the setup, indicating that it is well suited for use as a portable system outside the laboratory. This is confirmed as the figure shows that the overall setup can easily be arranged on a small (~80 cm × 45 cm) desk.

The spectrometer used was accessed using MATLAB code written by the authors, with an integration time of 400 ms being chosen (after some trial and error testing to enable a satisfactory signal level under ambient light conditions, without saturating the spectrometer) and the fluorescent spectrum was monitored over the wavelength range from 475 to 770 nm (with a resolution of 1.4 nm). 

Toggling of the LED was toggled by sending a pulse to an electromechanical relay using the spectrometer and the ‘dark spectrum’ was collected. This was subtracted digitally from the ‘bright spectrum’ to remove the effects of any interference due to the ambient light, thus enabling the sensor to be used effectively in the prevailing ambient light conditions. The mean of 10 recorded values each was used to create the data set employed in the determination of the pH value of the solution. (The source code developed is provided in the supplementary material.)

## 4. Results and Discussions

### 4.1. Characterization of the Optical Fibre Probe in a Static Liquid Sample

**Figure 4:**
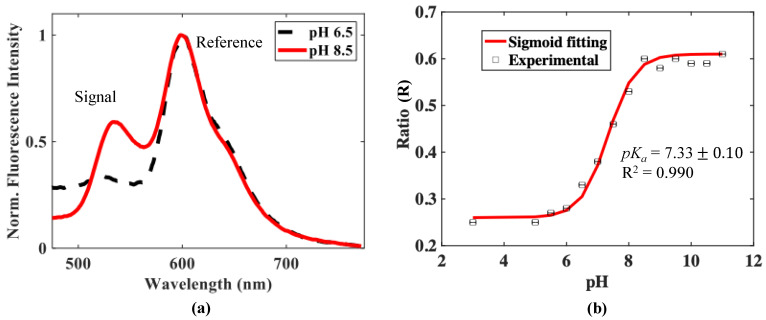
(**a**) Typical fluorescence curve showing two peaks corresponding to AAF dye and perylene red at the excitation wavelength of 375 nm for pH 6.5 and pH 8.5. The spectrum is normalized with respect to the reference peak. (**b**) The change in the ratio of signal to reference peak versus pH.

The typical fluorescence response of the sensor exhibiting two peaks, on excitation with light from a 375 nm LED source for pH 6.5 and pH 8.5 is shown in Figure 4a. The first peak, centred at a wavelength of ~534 nm, arises due to the AAF dye, and this signal is the one that is responsive to the external pH changes—hence, it is termed the ‘signal peak’. The second peak, centred at ~600 nm, is due to the perylene red. Since it is less responsive to any change in the external pH, it can be used to create an optical ‘reference signal’ in this way to allow for other non-pH-based fluctuations to be corrected. The large Stokes shift of the fluorescent peaks reduces the interference from light signal and allows for accurate measurements to be made without the need for any optical filters.

The Ratio (R) of the intensities of signal and reference peaks, which is the pH-dependent quantity (as given in Equation (4)), monitored for the change in the pH from a value of 3.0 to 11, is plotted in Figure 4b. It can be seen from the figure that the sensor showed a negligible change in response at low pH values, i.e., up to pH 6.0; however, then, the response increases approximately linearly with pH increases before saturating again, thus, showing an overall “S” shaped response. 

Based on this result, the effective working pH range of this sensor can be defined as from pH 6.0 to 8.5, a range useful for many applications, including for the survival of aquatic life—which thrives in this pH range and beyond which, disturbance is seen to the physiological systems of any organisms [65]. This sigmoid response is similar to that for a low pH sensor, as reported in previous work by some of the authors, where the working pH range was less, 0.5 to 6.0 [39]. This new sensor, while extending the range of pH measurements, is thus complementary in its response to that previously developed device. The combination of these devices thus allows a wide range of pH measurements, from 0.5 to 8.5, using either two individual probes or integrating their essential components in a combined probe.

On fitting Equation (6) to the experimental data obtained, it can be seen that there is a good agreement (R^2^ = 0.990) as shown in Figure 4b. The value obtained for *pK_a_*, 7.33 ± 0.1, which was determined from the fitting process, represents the value of pH at which 50% of the dye population in the solution is protonated.

The maximum sensitivity of the sensor using Equation (7) is found to be 0.2/pH unit, at a value of pH = 7.5. The *R_min_* and *R_max_* values were taken at the pH values 3 and 11 and *pKa* were taken as 7.33 in static liquid.

(7) $$\frac{dR}{dpH}=\frac{10^{\left(pH-pK_{a}\right)}\times \ln\left(10\right)\times \left(R_{max}-R_{min}\right)}{{\left(10^{\left(pH-pK_{a}\right)}+1\right)}^{2}}$$

**Figure 5:**
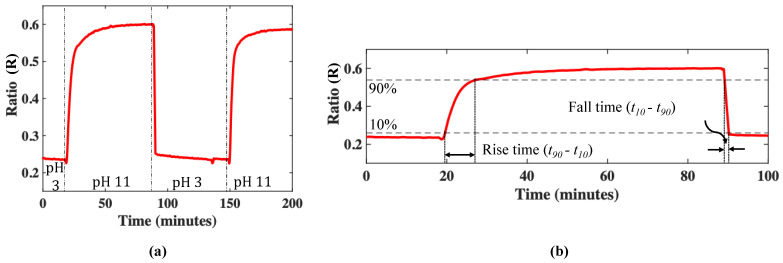
(**a**) The cyclical response of the sensor at two pH values 3 and 11. The vertical dotted-dashed lines show the pH change time. (**b**) The first 100 min of the sensor response shows the rise and fall times, *t_10_* and *t_90_*, which, respectively, represent the time taken to reach 10% of the lower and 90% of the higher value of the measured pH.

The repeatability of the sensor scheme thus developed was studied by measuring its cyclical response, with two extreme pH values, these being 3 and 11. Figure 5a shows the consistency of the response of the sensor. Figure 5b shows the rise time (*t_90_ – t_10_*) and fall time (*t_10_* – *t_90_*) of this sensor. From this graph, these are determined to be as follows: the rise time and fall times were 5.93 ± 0.94 min and 1.25 ± 0.17 min, respectively. This maximum response time is better than of some previously reported sensors (e.g., Wallace et al.), which report a response time of ≈8.33 min [66]. However, from a general sensor perspective, the rise time measured in this way is high—this also can be seen in comparison to earlier reported work by some of the authors on a low-value pH sensor, where the (rising) response time was ~25 s [39]. 

It seems likely that the rise time is affected by the greater thickness of the sensing layer (from that used in previous work performed), its affinity to water and the porosity of the coating. The thickness of the sensing layer is estimated to be around 3 mm. The thicker the layer, the stronger the signal but the longer the response time. Different thicknesses have been used depending on the applications. In this work, a stronger signal is more important to evaluate the performance of the system. The porosity of the layer was not the focus of this work. 

The polymer is hydrophilic and interacts well with water. The thickness of the coating used on a probe inevitably created a ‘trade-off’, usually involving sensitivity, speed of response, stability and durability. Thus, while a thicker, sensitive coating on the fibre is desirable to provide stability and to prevent damage, the high response time seen in the static liquid may be too long for some measurement situations. To achieve that trade-off requires further optimization of the probe, and this will be investigated in future work (with a view to its reduction by minimizing the sensor coating thickness yet functioning in a way commensurate with a satisfactory performance)—in the longer term, a lower rise time is sought to more closely match the sensor fall time.

The advantage of cross-selectivity can be seen from the pH sensors, which have been designed around the protonation–deprotonation mechanism in the aqueous solution. Unlike other chemical sensors where cross-selectivity is a critical issue (that often affects the successful application of the system), sensors based on the protonation–deprotonation mechanism are not affected by the presence of other species since the only parameter that causes a shift in the acid–base equilibrium is the pH change. It may be argued that ionic strength can affect *pKa* values, thus, resulting in errors in pH determination. However, previous studies by the authors have shown that this type of polymer sensor has no sensitivity to ionic strength, even at high concentrations of NaCl [39].

### 4.2. Characterization of the Optical Fibre Probe in a Microfluidic Channel with Fluid Flow

**Figure 6:**
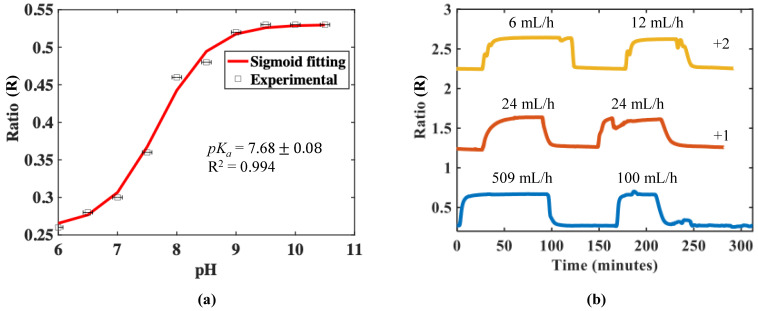
(**a**) The experimental and sigmoid curve fitting showing the change in the ratio (of signal to reference peak as a function of change in pH. (**b**) Cyclical response of sensor on three consecutive days with different flow rates. The pH varied from 3 to 11 and back. (Note: the red and yellow curves have been shifted in the y-direction by +1 and +2, respectively, for clarity.).

After characterizing the sensor in a static liquid, its response was measured in a microfluidic channel with various flow rates being used. All data were taken after the device was primed and was free of bubbles. Priming was done filling the channels at a slower flow rate of circa 500 µL/min. This allows the liquid to absorb any trapped air along the internal walls, preventing the formation of trapped bubbles. The change in the fluorescence ratio, R (as described above) and monitored as a function of the change in pH, is shown in Figure 6a. 

As can be seen from the figure, the sensor showed the expected “S-shaped” response and the experimentally determined performance matches well with that described by Equation (6), with R^2^ = 0.994. The value of *pK_a_* obtained was 7.68 ± 0.08, which is also close to that of the observed value in the static liquid (*pK_a_* = 7.33 ± 0.1). The maximum sensitivity of the sensor in the microfluidics using Equation (7) was found to be 0.16/pH unit, at a value of pH = 7.5. This result provides positive confirmation that a sensor of this design can be used effectively for pH measurements in a microfluidic channel, with a flowing, millilitre volume of liquid.

A syringe containing a pre-determined pH solution was used to allow the dye to be pumped into the inlet during experiments. Solutions of varying pH were syringe-pumped into the channel separately, with the channel being washed and dried after every solution. This was performed to ensure that the probe is only reading the pH of interest. At the lowest volumetric flowrate used of 6 mL/h, the flow velocity resulting from the dimensions of the rectangular inlet channel was calculated to be approximately 0.08 m/s (assuming the density of water = 998 kg/m^3^). With the distance from the inlet to the probe measuring around 250 mm, the slowest time required for the solution to reach the probe was estimated to be circa 3 s. For the highest volumetric flowrate used in Figure 6b of 509 mL/h, the solution will reach the probe in approximately 0.03 s.

The repeatability and time response of the sensor in the microfluidic channel arrangement was measured over the range from pH 3 to pH 11, on three consecutive days, with different flow rates being used as shown in Figure 6b. On the same day of measurement, as well as across several subsequent days, the response of the sensor was highly repeatable. 

However, the response time of the sensor was seen to be dependent on the flow rate used. In general, the response rise time seems to reduce with increased flow rate, likely due to the constant refreshing of hydrogen ions around the sensor head, increasing the availability of hydrogen ions reaching the sensor, as opposed to sensing in a static condition or at lower flow rates. The exchange of ions near the active area of the probe itself affects the protonation and deprotonation rate of the fluorescent dye. The active sensing area in contact with the liquid is approximately 3 mm^2^ in a 1 cm diameter well of approximately 30 µL in volume. 

Due to the location and position (on the top of the flow channel and in the middle) of the sensor tip, bubble formation around the sensor is unlikely once the channel has been properly primed. Changing the cylindrical hole pattern of the microfluidic channel (used for integrating the fibre) to an inverted funnel-shaped design might allow for more liquid to be in contact with the sensor head might resolve this, as the latter design will allow more solutions to come in contact with the sensor head. The exact reason for the presence of small oscillation in the signal in Figure 6b is still unclear and the subject of ongoing work: however, it does not create a major influence on the measurements made.

### 4.3. Performance Comparison with Previously Reported Optical Chemical pH Sensors

A comparison of the performance of the sensor developed in this work with several representatives and previously reported laboratory-based and commercially available optically-based chemical pH sensors is shown in Table 1. With the exception of the commercial sensors, most of the pH sensors reported in Table 1 lend themselves to integration into the design of most microfluidic platforms due to their size and currently reported manufacturing methods. However, it is important to note that most of the available, commercial optical chemical sensors are not readily compatible with microfluidics channels, and thus the development of such sensors is still an area of active research.

It can be seen from the table that the response time of the current sensor is somewhat higher; however, the real strength of this sensor lies in its portability, use of ratiometric detection, ease of integration with microfluidics (thus reducing the fabrication complexities), ease of multiplexing and ability to work in the ambient light. As stated earlier, current ongoing work seeks to reduce the response time by optimizing the thickness of the film and by changing the design of the microfluidic channel, as well also by increasing the pH working range by changing or multiplexing the dye used, such as to a coumarin dye (working pH range: 0.5–6.0) [36]. Optimizing this aspect is part of ongoing work. The estimated average precision of the measurement (see Figure 6a) of pH is ~ ± 0.2 pH units (from the data reported).

## 5. Conclusions

In the research we conducted, the pH-dependent fluorescence intensity of acrylamidofluorescein dye was exploited to develop a portable optical fibre-based pH sensor, and its response was studied in static liquid as well as in the dynamic flow conditions of the microfluidic channel—for which it was particularly suited. The results show that the sensor developed can be used both in a static measurement situation and in a microfluidic channel with an active flowrate. 

For the sensor scheme, with the dye used, the current optimum working pH range (of 6.0–8.5) is within the maximum range of 3–11 pH units; however, the pH range can be easily altered by changing the dye used, such as coumarin dye (working pH range: 0.5–6.0), or can be used in parallel (multiplexed on a single optical fibre) with other sensors working on same principles to cover a wider pH range if needed. 

Importantly, the portable optical fibre sensor developed can be easily integrated (and then separated from) with the microfluidic channel, without destroying either and allowing easy cleaning and reuse—in this way, reducing the cost of ownership and thus opening the door for its usage in a range of ‘real-life’ applications demonstrating that an accurate in-situ evaluation of pH is possible in a standard microfluidic device that is applicable for a variety of future applications.

## Figures and Tables

**Table 1:** Performance comparison with some previously reported lab-based or commercial optical chemical sensors.

Chemical Used/ Manufacturer	Detection Method	Ph Range	Key Specifications	Comments	Reference
Acrylamidofluorescein (AAF) dye	Intensity ratiometric method	6.0–8.5	Sensitivity: 0.2/pH; Response time < 6 min	Demonstrated for both static liquid as well as a microfluidic channel; does not need fluorescent liquid for detection.	This work
Coumarine dye	Intensity Change	0.5–6.0	Response time = 25 s	Measurement performed in the static liquid	[39]
Phenol red	Absorption	7.0–8.0	Accuracy: 4% in the working pH range Response time ≈ 10 s	Non-invasive method demonstrated for measuring pH in the microfluidics chamber but works only with liquid containing phenol red solution	[44]
8-hydroxypyrene-1,3,6-trisulfonic acid trisodium salt (HPTS)	Fluorescence emission	2.5–9	Response time = 10 s	PMMA, Bragg grating-based device. Static mixer for effective mixing of the indicator dye and the test solution	[46]
Bromothymol Blue	UV/visible Absorbance	2.7–10.5	Sensitivity = 0.04/pH Limit of detection = 1.58 ± 0.01 µM	New sensor material presented. Pre-mixed solution. Optical transmission from the waveguides is determined by absorbance. The quality of channels affects the coupling of the waveguide to the device.	[47]
m-Cresol Purple	Absorption	7.5–8.2	Detection time = 8 min Resolution = 0.002 pH units	Device for seawater pH measurement. Inline mixing channel to improve the homogeneity of seawater and indicator.	[48]
Phenol Red	Absorption	4–10	Response time = 15 s	Glass microcell. Pre-mixed solutions syringed to cell.	[49]
Phenol red	Absorption	6–8.5	Signal stable after 2 min	Chlorine-based pH sensor. Glass-based device. A linear relationship was not observed when pH was below 6 or 8.5	[50]
Chitosan Hydrogel	Effective optical thickness	6.2–7.4	Response time = 1.5 min (microfluidic) & 13 min (microfluidic)	Measured swelling of the Chitosan layer.	[67]
PreSens (Commercial)	Dual lifetime referencing	5.5–8.5	Resolution: At pH = 7.000 ± 0.001 pH Response time <2 min	Sensor patches, non-invasive. Not applied to microfluidic channels	[68]
Ocean Insight (Commercial)	Colorimetric	5.0–9.0	Resolution = 0.02 pH Response time = 30 s	Sensor patches, non-intrusive measurement. Cannot be used with coloured or turbid liquids. Yellow liquids can be used if the 750 nm baseline is used. Not applied to microfluidic channels,	[69]
Scientific Industries (Commercial)	Dual Excitation Ratiometric	5.5–8.0	Accuracy: 1.5% at pH 7 Response time < 15 s	Sensor patches, non-invasive measurement. Same system can also be used to measure dissolved oxygen in the liquid. Not applied to microfluidic channels.	[70]
m-Cresol Purple	Absorption	3.0–6.0	Response time = 90 s	With artificial neural network (ANN) to read out pH values in real-time. pH sensing membrane onto the wall of the microfluidic chamber.	[71]
PreSens (Commercial)	pH Microsensor	5.5–8.5	Response time at 25 °C ≤ 30 sec Resolution at pH = 7.00 ± 0.02 pH	pH-sensitive (swelling) coating -HP5	[72]
Poly (ethylene glycol) diacrylate (PEGDA)	Lateral stress inducing wavelength shift	1.0–6.5	Response time = 30 s Sensitivity = 0.41 nm/pH	pH-sensitive hydrogel on fibre Bragg grating	[73]
Bromophenol blue/Cresol red/Phenol red/phenolphthalein	Evanescent wave absorption	3.0–11.0	Response time = 0.05 s at pH 12 & 1.8 s at pH 3 Sensitivity = 7.65 counts/pH	Silica-titania on sol-gel	[74]
Polyaniline coating	Refractive index change	2.0–12.0	Maximum sensitivity = 82 pm/pH and a minimum of 30 pm/pH. Stabilisation at 6 pH = 29 s	pH-sensitive film on tilted fibre Bragg grating. Sensitivity is directly related to the film thickness	[75]
Polyaniline (PANI)-polyvinyl alcohol (PVA) Composite layer	Light absorption	2.0–9.0	Sensitivity of 2.79 µW for 2–9 pH	pH-sensitive with PANI-PVA composite film as a stimuli-responsive layer. pH-responsive changes in absorption properties due to changes in molecular conformation.	[76]

## Supplementary Materials

The following supporting information can be downloaded at: https://www.mdpi.com/article/10.3390/mi13081224/s1, MATLAB Source-Code.

## References

[1] Werner J., Belz M., Klein K.F., Sun T., Grattan K.T.V. (2021). Fiber optic sensor designs and luminescence-based methods for the detection of oxygen and pH measurement. Measurement.

[2] Yue Y., Huo F., Lee S., Yin C., Yoon J. (2017). A review: The trend of progress about pH probes in cell application in recent years. Analyst.

[3] Alam A.U., Qin Y., Nambiar S., Yeow J.T., Howlader M.M., Hu N.X., Deen M.J. (2018). Deen, Polymers and organic ma-terials-based pH sensors for healthcare applications. Prog. Mater. Sci..

[4] Swietach P., Vaughan-Jones R.D., Harris A.L., Hulikova A. (2014). The chemistry, physiology and pathology of pH in cancer. Philos. Trans. R. Soc. B Biol. Sci..

[5] Edgar W.M. (1976). The Role of Saliva in the Control of pH Changes in Human Dental Plaque. Caries Res..

[6] Carlsson S., Wiklund N.P., Engstrand L., Weitzberg E., Lundberg J.O.N. (2001). Effects of pH, Nitrite, and Ascorbic Acid on Nonenzymatic Nitric Oxide Generation and Bacterial Growth in Urine. Nitric Oxide.

[7] Kress-Rogers E. (1991). Solid-state pH sensors for food applications. Trends Food Sci. Technol..

[8] Singh H., Fox P.F. (1985). Heat stability of milk: pH-dependent dissociation of micellar k-casein on heating milk at ultra-high temperatures. J. Dairy Res..

[9] Domingues D.S., Takahashi H.W., Camara C.A., Nixdorf S.L. (2012). Automated system developed to control pH and concentration of nutrient solution evaluated in hydroponic lettuce production. Comput. Electron. Agric..

[10] von Bültzingslöwen C., McEvoy A.K., McDonagh C., MacCraith B.D., Klimant I., Krause C., Wolfbeis O.S. (2002). Sol–gel based optical carbon dioxide sensor employing dual luminophore referencing for application in food packaging technology. Analyst.

[11] Van der Schueren L., De Clerck K. (2010). The use of pH-indicator dyes for pH-sensitive textile materials. Text. Res. J..

[12] Van der Schueren L., De Clerck K. (2012). Coloration and application of pH-sensitive dyes on textile materials. Color. Technol..

[13] EPA (2001). Parameters of Water Quality: Interpretations and Standards.

[14] Yu H.Q., Fang H.H.P. (2002). Acidogenesis of dairy wastewater at various pH levels. Water Sci. Technol..

[15] Boczkaj G., Fernandes A. (2017). Wastewater treatment by means of advanced oxidation processes at basic pH conditions: A review. Chem. Eng. J..

[16] Poloczanska E.S., Brown C.J., Sydeman W.J., Kiessling W., Schoeman D.S., Moore P.J., Brander K., Bruno J.F., Buckley L.B., Burrows M.T. (2013). and Richardson, A.J. Global imprint of climate change on marine life. Nat. Clim. Chang..

[17] Devau N., Le Cadre E., Hinsinger P., Jaillard B., Gérard F. (2009). Gérard, Soil pH controls the environmental availability of phos-phorus: Experimental and mechanistic modelling approaches. Appl. Geochem..

[18] Poma N., Vivaldi F., Bonini A., Carbonaro N., Di Rienzo F., Melai B., Kirchhain A., Salvo P., Tognetti A., Di Francesco F. (2019). Remote monitoring of seawater temperature and pH by low cost sensors. Microchem. J..

[19] Zhang C., van Noort D. (2011). Cells in microfluidics. Top Curr. Chem..

[20] Manjakkal L., Dervin S., Dahiya R. (2020). Flexible potentiometric pH sensors for wearable systems. RSC Adv..

[21] Kraikaew P., Jeanneret S., Soda Y., Cherubini T., Bakker E. (2020). Ultrasensitive Seawater pH Measurement by Capacitive Readout of Potentiometric Sensors. ACS Sens..

[22] Perumal V., Prasad R.H., Hashim U. pH Measurement using in house fabricated interdigitated capacitive transducer. Proceedings of the RSM 2013 IEEE Regional Symposium on Micro and Nanoelectronics.

[23] Arefin S., Coskun M.B., Alan T., Neild A., Redoute J.-M., Yuce M.R. A MEMS capacitive pH sensor for high acidic and basic solutions. Proceedings of the Sensors.

[24] Wang T., Green R., Guldiken R., Mohapatra S., Mohapatra S. (2019). Multiple-layer guided surface acoustic wave (SAW)-based pH sensing in longitudinal FiSS-tumoroid cultures. Biosens. Bioelectronic..

[25] Oh H., Lee K.J., Baek J., Yang S.S., Lee K. (2013). Development of a high sensitive pH sensor based on shear horizontal surface acoustic wave with ZnO nanoparticles. Microelectron. Eng..

[26] Paul D., Dutta S., Saha D., Biswas R. (2017). LSPR based Ultra-sensitive low cost U-bent optical fiber for volatile liquid sensing. Sens. Actuators B Chem..

[27] Saikia R., Buragohain M., Datta P., Nath P., Barua K. (2009). Fiber-Optic pH Sensor Based on SPR of Silver Nanostructured Film. AIP Conference Proceedings.

[28] Toh Y.R., Yu P., Wen X., Tang J., Hsieh T.S. (2013). Induced pH-dependent shift by local surface plasmon resonance in functionalized gold nanorods. Nanoscale Res. Lett..

[29] Mishra S.K., Gupta B.D. (2013). Surface plasmon resonance based fiber optic pH sensor utilizing Ag/ITO/Al/hydrogel layers. Analyst.

[30] Singh S., Gupta B.D. (2012). Fabrication and characterization of a highly sensitive surface plasmon resonance based fiber optic pH sensor utilizing high index layer and smart hydrogel. Sens. Actuators B Chem..

[31] Nielsen L.J., Eyley S., Thielemans W., Aylott J.W. (2010). Dual fluorescent labelling of cellulose nanocrystals for pH sensing. Chem. Commun..

[32] Hecht M., Kraus W., Rurack K. (2013). A highly fluorescent pH sensing membrane for the alkaline pH range incorporating a BODIPY dye. Analyst.

[33] Gou P., Kraut N.D., Feigel I.M., Bai H., Morgan G.J., Chen Y., Tang Y., Bocan K., Stachel J., Berger L. (2014). Carbon Nanotube Chemiresistor for Wireless pH Sensing. Sci. Rep..

[34] Maroto A., Balasubramanian K., Burghard M., Kern K. (2007). Functionalized Metallic Carbon Nanotube Devices for pH Sensing. ChemPhysChem.

[35] Juang Y.-Z., Lin C.-F., Tsai H.-H., Liao H.-H., Wang R.-L. (2012). CMOS Biomedical Sensor with In Situ Gold Reference Electrode for Urine Detection Application. Procedia Eng..

[36] Surre F., Lyons W.B., Sun T., Grattan K.T.V., O’Keeffe S., Lewis E., Elosua C., Hernaez M., Barian C. (2009). U-bend fibre optic pH sensors using layer-by-layer electrostatic self-assembly technique. J. Physics Conf. Ser..

[37] Sharma N.K., Gupta B.D. (2003). Fabrication and characterization of pH sensor based on side polished single mode optical fiber. Opt. Commun..

[38] Nguyen T.H., Tong S. (2020). Optical Fibre Chemical Sensors, Optical Fibre Sensors: Fundamentals for Development.

[39] Nguyen T.H., Venugopalan T., Sun T., Grattan K.T.V. (2015). Intrinsic Fiber Optic pH Sensor for Measurement of pH Values in the Range of 0.5–6. IEEE Sens. J..

[40] Zheng Y., Chen L.H., Dong X., Yang J., Long H.Y., So P.L., Chan C.C. (2015). Miniature pH Optical Fiber Sensor Based on Fabry–Perot Interferometer. IEEE J. Sel. Top. Quantum Electron..

[41] Corres J.M., Matias I.R., del Villar I., Arregui F.J. (2007). Design of pH Sensors in Long-Period Fiber Gratings Using Polymeric Nanocoatings. IEEE Sens. J..

[42] Zubiate P., Zamarreño C.R., Del Villar I., Matias I.R., Arregui F.J. D-shape optical fiber pH sensor based on Lossy Mode Resonances (LMRs). Proceedings of the Sensors.

[43] Tough T. https://www.turtletoughsensors.com/products/liquid-analysis/ph-orp.

[44] Magnusson E.B., Halldorsson S., Fleming R.M.T., Leosson K. (2013). Real-time optical pH measurement in a standard micro-fluidic cell culture system. Biomed. Opt. Express.

[45] Liao Z., Zhang Y., Li Y., Miao Y., Gao S., Lin F., Deng Y., Geng L. (2019). Microfluidic chip coupled with optical biosensors for simultaneous detection of multiple analytes: A review. Biosens. Bioelectron..

[46] Moradi V., Akbari M., Wild P. (2019). A fluorescence-based pH sensor with microfluidic mixing and fiber optic detection for wide range pH measurements. Sens. Actuators A Phys..

[47] Zamboni R., Zaltron A., Izzo E., Bottaro G., Ferraro D., Sada C. (2020). Opto-Microfluidic System for Absorbance Measurements in Lithium Niobate Device Applied to pH Measurements. Sensors.

[48] Pinto V., Araújo C., Sousa P., Gonçalves L., Minas G. (2019). A low-cost lab-on-a-chip device for marine pH quantification by colorimetry. Sens. Actuators B Chem..

[49] Budinski V., Donlagic D. (2021). All Silica Micro-Fluidic Flow Injection Sensor System for Colorimetric Chemical Sensing. Sensors.

[50] Elmas S., Pospisilova A., Sekulska A.A., Vasilev V., Nann T., Thornton S., Priest C. (2020). Photometric Sensing of Active Chlorine, Total Chlorine, and pH on a Microfluidic Chip for Online Swimming Pool Monitoring. Sensors.

[51] Sieben V.J., Floquet C.F.A., Ogilvie I.R.G., Mowlem M.C., Morgan H. (2010). Microfluidic colourimetric chemical analysis system: Application to nitrite detection. Anal. Methods.

[52] Al-Mugahiry B., Al-Lawati H.A.J. (2020). Recent analytical advancements in microfluidics using chemiluminescence detection systems for food analysis. TrAC Trends Anal. Chem..

[53] Tiwari S., Bhat S., Mahato K.K. (2020). Design and Fabrication of Low-cost Microfluidic Channel for Biomedical Application. Sci. Rep..

[54] Sackmann E.K., Fulton A.L., Beebe D.J. (2014). The present and future role of microfluidics in biomedical research. Nature.

[55] Yang Y., Chen Y., Tang H., Zong N., Jiang X. (2020). Microfluidics for Biomedical Analysis. Small Methods.

[56] Zhao Y., Hu X.-G., Hu S., Peng Y. (2020). Applications of fiber-optic biochemical sensor in microfluidic chips: A review. Biosens. Bioelectron..

[57] Choi S., Goryll M., Sin L.Y.M., Wong P.K., Chae J. (2011). Microfluidic-based biosensors toward point-of-care detection of nucleic acids and proteins. Microfluid. Nanofluidics.

[58] Mark D., Haeberle S., Roth G., Stetten F.V., Zengerle R. (2010). Microfluidic lab-on-a-chip platforms: Requirements, characteristics and applications. Microfluidics Based Microsystems.

[59] Mukhopadhyay S. (2020). Short Review on Microfluidic Lab-on-a-Chip Systems for Future Applications in Space Technology. J. Nanosci. Nanoeng. Appl..

[60] Okazaki T., Watanabe T., Kuramitz H. (2020). Evanescent-Wave Fiber Optic Sensing of the Anionic Dye Uranine Based on Ion Association Extraction. Sensors.

[61] Thangaraj S., Paramasivan C., Balusamy R., Arumainathan S., Thanigainathan P. (2020). Evanescent wave optical fibre ammonia sensor with methylamine hydroiodide. IET Optoelectron..

[62] Dhara P., Kumar R., Binetti L., Nguyen H.T., Alwis L.S., Sun T., Grattan K.T.V. (2019). Optical Fiber-Based Heavy Metal Detection Using the Localized Surface Plasmon Resonance Technique. IEEE Sens. J..

[63] Munkholm C., Parkinson D.R., Walt D.R. (1990). Intramolecular fluorescence self-quenching of fluoresceinamine. J. Am. Chem. Soc..

[64] Johnston I.D., Tracey M.C., Davis J.B., Tan C.K.L. (2005). Micro throttle pump employing dis-placement amplification in an elastomeric substrate. J. Micromech. Microengin..

[65] Sansalvador I.M.P.D.V., Fay C., Cleary J., Nightingale A., Mowlem M.C., Diamond D. (2016). Autonomous reagent-based microfluidic pH sensor platform. Sens. Actuators B Chem..

[66] A Wallace P., Elliott N., Uttamlal M., Holmes-Smith A.S., Campbell M. (2001). Development of a quasi-distributed optical fibre pH sensor using a covalently bound indicator. Meas. Sci. Technol..

[67] Tang Y., Zhen L., Liu J., Wu J. (2013). Rapid Antibiotic Susceptibility Testing in a Microfluidic pH Sensor. Anal. Chem..

[68] PreSens pH Sensors, (n.d.). https://www.presens.de/products/detail/ph-sensor-spots-sp-hp5.

[69] Ocean Insight pH Sensor Manual, (n.d.). https://www.oceaninsight.com/globalassets/catalog-blocks-and-images/manuals--instruction-old-logo/sensors/ph-sensors-patches-probes-and-cuvettes.pdf.

[70] CellPhase System, (n.d.). https://www.scientificindustries.com/bioprocessing/cellphase/cellphase-system.html.

[71] Lu Y., Feng Q., Zhang R., Lu H., Su J., Cui Y., Zhu L. (2020). An online pH detection system based on a microfluidic chip. Anal. Chim. Acta.

[72] PreSens pH Sensors, (n.d.). https://www.presens.de/products/detail/implantable-ph-microsensor-imp-hp5.

[73] Cheng X., Bonefacino J., Guan B.-O., Tam H.Y. (2018). All-polymer fiber-optic pH sensor. Opt. Express.

[74] Islam S., Bidin N., Riaz S., Krishnan G., Naseem S. (2016). Sol–gel based fiber optic pH nanosensor: Structural and sensing prop-erties. Sens. Actuators A Phys..

[75] Aldaba A.L., González-Vila Á., Debliquy M., Lopez-Amo M., Caucheteur C., Lahem D. (2018). Polyaniline-coated tilted fiber Bragg gratings for pH sensing. Sens. Actuators B Chem..

[76] Khanikar T., Singh V.K. (2019). PANI-PVA composite film coated optical fiber probe as a stable and highly sensitive pH sensor. Opt. Mater..

