# Supplementary material for: A Portable ‘Plug-and-Play’ Fibre Optic Sensor for In-Situ Measurements of pH Values for Microfluidic Applications
Source: Micromachines (Basel). 2022 Jul 30;13(8):1224. doi: 10.3390/mi13081224 (PMC9416338; doi:10.3390/mi13081224)
Supplement: Supplementary file 1 [file micromachines-13-01224-s001.zip › micromachines-1824121-supplementary.pdf]

Supplementary Materials:

Matlab Source-Code

```
close all; clearvars; clc

% Plz refer Ocean Optics programming module for spectrometer related commands
spectrometerIndex = 0;
Integration_time = 400000; % integration time

% array to store and display ratio of two fluorescent peaks obtained for 100 time steps
ratio_reading = zeros(1,100);

%counter to keep track of the ratio, i.e. 100 time steps
i = 1;

% opening the two files one for stroing spectrum and ratio
Filename = '<file_name>.csv';
Filename1 = sprintf('file_name1_%s.csv', datestr(now,'mm-dd-yyyy HH-MM-SS'));
fid = fopen(Filename, 'a');
fid1 = fopen(Filename1, 'a');
% writing header
fprintf(fid,'%s, %d\n','Integration_time (in sec) =',Integration_time/10^6);
fprintf(fid, 'Time Stamp (HH-MM-SS), Raw_ratio, Ratio_smooth\n');

% accessing driver of the spectrometer
try
   handles.wrapper = com.oceanoptics.omnidriver.api.wrapper.Wrapper();
catch
   javaaddpath('C:\Program Files\Ocean Optics\OmniDriver\OOI_HOME\OmniDriver.jar');
   handles.wrapper = com.oceanoptics.omnidriver.api.wrapper.Wrapper();
end

handles.wrapper.openAllSpectrometers();

handles.wavelengths = handles.wrapper.getWavelengths(0); %get wavelengths to form the X axis
handles.wrapper.setIntegrationTime(0,Integration_time);

% capturing dark spectrum
handles.wrapper.setStrobeEnable(spectrometerIndex,0); %send signal via spectrometer to turn off LED (pin 13)
handles.wrapper.getSpectrum(0); %get inetesity counts for each wavelength
SpecDark=handles.wrapper.getSpectrum(0);

% capturing bright spectrum
handles.wrapper.setStrobeEnable(spectrometerIndex,1); %send signal via spectrometer to turn on LED
handles.wrapper.getSpectrum(0);
SpecOn = handles.wrapper.getSpectrum(0);


SpecResult=SpecOn-SpecDark; % spectrum after removing ambient light (dark) component from the bright one.
smooth_SpecResult_1 = smooth(handles.wavelengths,SpecResult,0.015,'loess');
```

```
% writing spectrum to the file
fprintf(fid1,'%f, %f\n',handles.wavelengths,SpecResult);
fclose(fid1) ;

% plotting raw and smooth spectrum
figure()
k =
plot(handles.wavelengths,SpecResult,handles.wavelengths,smooth_SpecResu
lt_1);
k(1).LineWidth = 4;
k(1).Color = 'R';
k(2).LineWidth = 4;
k(2).Color = 'B';
set ( gca , 'FontSize' , 24 , 'fontweight' , 'b' , 'FontName' , 'Times New Roman') ;
xlabel ( 'Wavelength' , 'FontSize' , 24 , 'fontweight' , 'b' ,'FontName' , 'Times New
Roman') ;
ylabel ( 'Intensity  (a.u.)','FontSize' , 24 , 'fontweight' , 'b', 'FontName' , 'Times
New Roman' ) ;
xlim([450 780]);

% plotting the ratio and later using while loop to update it in  real time
figure()
Ra = plot(ratio_reading);
Ra(1).LineWidth = 4;
Ra(1).Color = 'R';
set ( gca , 'FontSize' , 24 , 'fontweight' , 'b' , 'FontName' , 'Times New Roman') ;
xlabel ( 'Iteration' , 'FontSize' , 24 , 'fontweight' , 'b' ,'FontName' , 'Times New
Roman') ;
ylabel ( 'Ratio','FontSize' , 24 , 'fontweight' , 'b', 'FontName' , 'Times New
Roman' ) ;
ylim([0 1]);

% loop to calculate the ratio and update the figure in real-time
while (ishandle(Ra))

  % check if array is full
  if i == 100
    i = 1;
    ratio_reading = zeros(1,100);
  end

  % capture dark spectrum
  handles.wrapper.setStrobeEnable(spectrometerIndex,0);
  handles.wrapper.getSpectrum(0);% getSpectrum should be called twice.
Please refer Ocean Optics manual
  SpecDark=handles.wrapper.getSpectrum(0); % get counts for each
wavelength

  % capture bright spectrum
  handles.wrapper.setStrobeEnable(spectrometerIndex,1); % pin 13
  handles.wrapper.getSpectrum(0);
  SpecOn=handles.wrapper.getSpectrum(0);

  SpecResult=SpecOn-SpecDark; % differencial on - dark to cancel the outside
light
  smooth_SpecResult = smooth(handles.wavelengths,SpecResult,0.015,'loess');


  [indref, ~]= find(handles.wavelengths(:,1) >= 596 & handles.wavelengths(:,1)
```

```
< 610);  % parse values of the ref peak
   [indsignal, ~]= find(handles.wavelengths(:,1) >= 524 &
handles.wavelengths(:,1) < 544); % parse values of the PH peak
   % raw data
   Ref=mean(SpecResult(indref));  % integrate counts on ref peak
   Signal=mean(SpecResult(indsignal));  % integrate counts on pH peak
   ratio_raw=Signal/Ref;

   % Ratio of the smoothened curve
   Ref_smooth=mean(smooth_SpecResult(indref));   %integrate counts on ref
peak
   Signal_smooth=mean(smooth_SpecResult(indsignal));  %integrate counts on
PH peak
   ratio_smooth=Signal_smooth/Ref_smooth;    %calculate ratio (must be
calibrated to right PH value)


   % storing the value in array
   ratio_reading(i) = ratio_smooth;

   % opening the file to write spectrum
   Filename1 = sprintf('file_name1_%s.csv', datestr(now,'mm-dd-yyyy HH-
MM-SS'));
   fid1 = fopen(Filename1, 'a');
   % wrtiting spectrum to file
   fprintf(fid1,'%f, %f\n',handles.wavelengths,SpecResult);
   % close the spectrum file
   fclose(fid1) ;

   % wrtiting the ratio to file with time_stamp
   fprintf(fid,'%s, %f, %f\n', datestr(now,'HH-MM-SS'), ratio_raw,
ratio_smooth);


   % increasing the counter by 1
   i = i+1;

   %updates the display
   set(Ra,'ydata',ratio_reading);
   set(k,'ydata',SpecResult)
   drawnow;
end

handles.wrapper.closeAllSpectrometers()
fclose(fid) ; % close the file used for writing ratio
```

Published with MATLAB® R2019b
